# Geopolymers as a Sustainable Chemical Platform: From Building Materials to Advanced Functional Applications

**DOI:** 10.1002/tcr.70191

**Published:** 2026-06-11

**Authors:** Martina Maria Calvino, Lorenzo Lisuzzo, Giuseppe Cavallaro, Stefana Milioto, Giuseppe Lazzara

**Affiliations:** ^1^ Dipartimento di Fisica e Chimica ‐ Emilio Segrè Università degli Studi di Palermo Palermo Italy

**Keywords:** alkali activation, aluminosilicate precursors, functional inorganic materials, geopolymer, halloysite nanotubes, sustainability

## Abstract

Geopolymers are aluminosilicate inorganic polymers traditionally investigated as sustainable alternatives to ordinary cement. This review rethinks geopolymers not merely as sustainable binders but as a versatile chemical platform whose properties arise from controllable dissolution–condensation pathways, nanoscale structural disorder, and activator chemistry. We critically analyze the sustainability considering life‐cycle assessments and discuss how activator production and curing conditions challenge oversimplified green claims in recent literature. The transition from bulk construction materials to nanoengineered and hybrid systems is examined, highlighting the role of nanofillers and interfacial engineering strategies. A particular focus is devoted to halloysite‐based systems, tracing their evolution from early nanoarchitectural surface modification of fly ash particles to recent developments in self‐assembled inorganic films with tunable morphology at a mesoscopic length scale. These systems demonstrate that geopolymerization can act as a structural converter across nano‐, meso‐, and macroscales, enabling applications in barrier, adsorption, CO_2_ capture, and environmental remediation. We conclude by outlining design principles for future geopolymer research, emphasizing activation chemistry, structural control, and morphological programmability as key aspects in developing sustainable functional materials for advanced applications. Future efforts will need to identify sustainable activation strategies for construction applications and to develop chemically adaptable systems for high‐performance functional uses.

## Graphical abstract

Geopolymers are presented as a chemically programmable platform whose properties emerge from activation chemistry, nanoscale disorder, and interfacial design. The review highlights sustainability challenges, the shift toward nanoengineered and hybrid systems, and the rise of halloysite‐based architectures enabling tunable structures across scales for barrier, adsorption, CO_2_ capture, and environmental remediation applications.

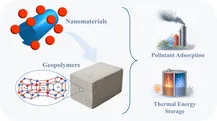

## 1 Introduction

Concrete is the most widely used synthetic material on Earth. Its production relies heavily on ordinary portland cement (OPC), whose manufacturing contributes approximately 5%–7% of global anthropogenic CO_2_ emissions [1, 2]. The environmental consequences associated with limestone calcination and high‐temperature brick formation have motivated extensive research into alternative building materials. Geopolymers are considered promising candidates in this context [3, 4, 5]. They are inorganic polymers based on aluminosilicate networks, and they are formed through the alkaline activation of silica‐ and alumina‐rich precursors such as fly ash, metakaolin, and slag [6, 7, 8, 9]. The geopolymerization process involves three interconnected steps: (i) dissolution of aluminosilicate species in alkaline solution, (ii) gelation through condensation reactions, and (iii) polymerization into a three‐dimensional (3D) network [10]. Geopolymeric systems are generally associated with low‐calcium precursors and lead to the formation of N–A–S–H type gels. In contrast, alkali activation of calcium‐rich precursors such as blast furnace slag results in markedly different reaction products, typically dominated by C–A–S–H or hybrid C–(N)–A–S–H gels. The presence of calcium significantly modifies the reaction mechanism, promoting faster kinetics and leading to distinct microstructures compared to low‐calcium systems [11]. As a result, while both classes of materials fall under the broader category of alkali‐activated materials, the term “geopolymer” is more strictly associated with calcium‐poor systems forming polysialate‐type networks. These compositional differences also translate into distinct durability behaviors, as calcium‐rich systems generally exhibit faster reaction kinetics and higher early strength but may show different long‐term stability and chemical resistance compared to low‐calcium geopolymer gels.

Recent reports consider geopolymers as sustainable cement replacements. However, comprehensive life‐cycle analyses have demonstrated that sustainability claims strongly depend on preparation protocols and raw material sources [12]. In particular, the production of sodium silicate and sodium hydroxide can account for most carbon dioxide emissions associated with geopolymer synthesis [13]. Thus, the sustainability of geopolymers cannot be reduced to the absence of limestone calcination. Instead, it depends on a broader chemical perspective that takes into account the precursor selection, activator sourcing, curing protocols, and end‐of‐life considerations. At the same time, the application of geopolymers can be extended beyond construction materials, namely, advances in nanostructuring, hybridization, and interfacial engineering have revealed that geopolymers possess characteristics of tunable inorganic polymer platforms also for performing coatings or functional bulk materials [14, 15, 16]. Recent studies demonstrate that geopolymer matrices can be engineered at the nanoscale through filler incorporation, controlled porosity, and hierarchical architectures [17, 18]. In parallel, halloysite‐based systems have opened new pathways in which preorganized natural nanotubes serve either as nanoarchitectural modifiers [19] or as active precursors in self‐assembled inorganic films [20] and hollow geopolymeric capsules [21]. These developments suggest that geopolymers should not be defined primarily by their application in construction but by their capacity for programmable inorganic network formation across length scales. Recent advances further broaden the scope of geopolymer research. Machine‐learning models have been applied to predict strength development in coal‐gangue‐based systems [22], while nanosilica has been shown to refine microstructure and enhance mechanical performance [23]. Interfacial engineering using acid or alkaline catalyzed nano‐SiO_2_ sols has improved bonding in hybrid OPC–geopolymer assemblies [24, 25], and life‐cycle studies confirm the viability of coal‐gangue‐based formulations as sustainable OPC alternatives [26]. Together, these developments highlight the growing convergence of nanomodification, interfacial control, and data‐driven design in next‐generation geopolymer technologies.

This review therefore adopts a platform‐oriented perspective. Rather than cataloging mechanical properties or durability metrics, we examine geopolymers through three central lenses: (1) chemical fundamentals and structural disorder; (2) sustainability as building material for large‐scale applications; and (3) morphological programmability and functional integration in smart or advanced technology applications. By integrating foundational reviews, life‐cycle assessments (LCAs), nanoengineering advances, and halloysite‐driven morphogenetic systems, we aim to define a coherent framework for the next generation of geopolymeric materials.

## 2 Geopolymers as Inorganic Polymers

### 2.1 Dissolution–Condensation Thermodynamics

Geopolymerization is fundamentally a dissolution‐driven polycondensation process occurring under highly alkaline conditions. The first step involves the hydrolytic breakdown of aluminosilicate precursors such as fly ash or metakaolin, releasing silicate and aluminate oligomers into solution. This dissolution is strongly dependent on pH, activator composition, temperature, and the structural order of the precursor material. As originally systematized in early state‐of‐the‐art reviews [10], the reaction sequence includes solubilization, speciation, gelation, and large‐scale polymerization. From a kinetic point of view, the dissolution rate (*v*_diss_) of the solid aluminosilicate phase can be expressed as a surface‐controlled process:



(1)

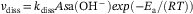

where *As* is the reactive surface area, *E*_a_ is the apparent activation energy, *R* is the gas constant, *T* is the temperature, and a(OH^−^) is the activity of hydroxyl ions, reflecting the strong dependence of dissolution on alkaline conditions. It represents the dependence of dissolution kinetics on alkalinity and is commonly described in literature through empirical or semiempirical relationships. Its exact form is system dependent and varies with precursor composition and solution chemistry. The Arrhenius‐type dependence and surface‐controlled kinetics of silicate dissolution have been extensively discussed in geochemical and cementitious systems [27, 28]. The dissolution step determines the concentration of reactive species such as H_3_SiO_4_^−^ and Al(OH)_4_^−^, which subsequently undergo polycondensation. Unlike Portland cement hydration, which is governed by dissolution–precipitation equilibria of crystalline and amorphous phases, geopolymerization generates an amorphous 3D aluminosilicate network. The thermodynamic landscape is therefore controlled by the stability of [SiO_4_] and [AlO_4_] tetrahedra under alkaline conditions and by charge compensation through cations such as sodium or potassium. The relative preference for Si–O–Al versus Si–O–Si linkages can be rationalized thermodynamically. Computational and spectroscopic studies indicate that, under alkaline activation, the formation of Si–O–Al bonds is often energetically favored relative to purely siliceous Si–O–Si linkages primarily due to electrostatic stabilization provided by alkali cations compensating the framework charge [29]. This energetic bias is consistent with Lowenstein's rule, which prohibits Al–O–Al linkages in aluminosilicate frameworks and promotes mixed Si–O–Al connectivity in geopolymer gels [30].

Dissolution and condensation are not strictly sequential but kinetically overlapping processes. Gelation is often the rate‐controlling step, and its kinetics can be described using an Avrami‐type expression:

(2) $$\alpha (t)=1‐exp(‐k_{g}t^{n})$$

where *α* is the degree of gel conversion, *k*_g_ is the gelation rate constant, and *n* is an exponent reflecting nucleation and growth mechanisms [31]. Avrami‐type kinetics have been applied to geopolymerization and related amorphous network formation processes [32].

In this framework, the Avrami parameters are used to describe gelation kinetics in geopolymer systems. The rate constant *k*_*g*_ reflects the overall kinetics of gel formation and is strongly influenced by precursor dissolution rate, alkalinity, and temperature, which control the availability of reactive silicate and aluminate species. The exponent *n* may reflect nucleation and growth mechanism of the gel phase, although its mechanistic interpretation in complex systems such as geopolymers is not univocally defined. In geopolymer systems, gel nucleation is typically heterogeneous. Nucleation occurs on partially dissolved precursor surfaces, and it is generally associated with polycondensation of silicate and aluminate species. As a result, deviations from ideal Avrami behavior are commonly observed in such complex reactive systems.

As gelation progresses, a cross‐linked inorganic polymer network forms. Within the framework of rubber elasticity theory adapted to network solids, the elastic modulus (E) can be related to the effective cross‐link density ν as E ≈ 3 ν RT.

In geopolymers, ν is governed by the Si/Al ratio, degree of condensation, and presence of secondary phases (e.g., C–(A)–S–H domains in Ca‐rich systems). The direct correlation between network connectivity and mechanical stiffness in alkali‐activated materials is crucial when discussing processability strategies. Setting time, viscosity evolution, and mechanical performance are emergent properties of dissolution rate, gelation kinetics, and cross‐link density development. Consequently, activation chemistry, temperature control, and precursor morphology become primary factors for tuning geopolymer synthesis and performances [33, 34].

The Si/Al ratio governs cross‐link density, mechanical rigidity, and chemical resistance [35]. Ratios typically between 1 and 3 produce mechanically stable materials, while higher ratios approach silica‐rich gel structures. This chemical flexibility underpins the concept of geopolymers as inorganic polymers rather than a simple cementitious binder.

### 2.2 Geopolymer–Zeolite Continuum and Structural Disorder

A critical conceptual advance in geopolymer science has been the recognition of continuity between amorphous geopolymer gels and crystalline zeolitic phases. Under certain curing conditions, partial ordering and nucleation of zeolite‐like domains can occur within the amorphous matrix. Under appropriate curing conditions, such as elevated temperature (up to 100°C), high Na concentration, and sufficient mobility of dissolved species, the initially amorphous aluminosilicate network may partially reorganize toward ordered domains, giving rise to zeolite‐like clusters within the gel matrix [36].

This geopolymer–zeolite continuum suggests that structural disorder is not a defect but a design parameter. Controlled disorder can tune mechanical strength, permeability resistance, or adsorption capacity, depending on the target application. Recent nanostructural investigations have reinforced this perspective by demonstrating that local ordering, nanoscale heterogeneity, and interfacial chemistry play decisive roles in determining macroscopic properties [37, 38]. Geopolymers should be therefore understood as charge‐balanced, alkali‐compensated aluminosilicate polymers whose properties emerge from controlled dissolution–condensation kinetics and structural disorder.

## 3 Sustainability: Chemical Realities

### 3.1 LCA and Activator Impact

The environmental appeal of geopolymers is frequently attributed to the absence of limestone calcination. However, detailed LCAs have demonstrated that activator production significantly influences total carbon footprint for geopolymeric materials [13]. Conventional OPC manufacturing typically generates up to 0.90 kg CO_2_ per kg of cement. In contrast, aluminosilicate precursors such as fly ash or slag are often associated with lower emissions when treated as industrial by‐products [39], although these benefits strongly depend on allocation methodology, regional availability, and resource constraints, particularly for slag [40]. However, detailed LCAs have demonstrated that this apparent advantage is highly sensitive to the contribution of alkaline activators [41]. Sodium hydroxide exhibits significant emission, while sodium silicate produced through high‐temperature fusion of silica sand and sodium carbonate (ca.1400°C) is also contributing to the total energy balance. Considering a typical fly ash‐based geopolymer formulations and in comparison to a conventional concrete, alkaline activators may account for 60%–75% of the total carbon emission of the geopolymer system, and the overall emission reduction relative to OPC concrete is below 10%. Recent LCA studies also show that the environmental performance of geopolymers varies significantly with precursor type. For example, global warming potentials reported for metakaolin‐, slag‐, and fly‐ash‐based systems range from approximately 1–4 kg CO_2_ eq., compared to the much higher reference value of OPC concrete. Other impact categories, including human toxicity and ecotoxicity, likewise exhibit strong precursor‐dependent variability, underscoring that the sustainability of geopolymers cannot be generalized and must be evaluated on a formulation‐specific basis [42]. Furthermore, elevated curing temperatures (e.g., 60°C–80°C for 12–24 h) can introduce an additional CO_2_ production. These quantitative comparisons demonstrate that sustainability of geopolymers cannot be assumed a priori. Only when low‐carbon or waste‐derived activation strategies are implemented, and ambient curing is feasible, the geopolymeric material consistently exhibits an environmental advantage [43].

### 3.2 Alternative Activation Pathways

Provided that alkaline activators may account for most of the total emissions in geopolymer systems, recent research has increasingly focused on replacing commercial sodium hydroxide and sodium silicate with waste‐derived or solid‐state activation pathways. One promising strategy involves exploiting the alkalinity of industrial residues such as red mud (bauxite residue) and calcium carbide slag [44]. Red mud typically contains 5–15 wt% Na_2_O (with respect to the dry precursor) and exhibits pH values between 10 and 13, enabling it to function both as an aluminosilicate precursor and as a partial alkali source. Calcium carbide slag, composed primarily of Ca(OH)_2_ (>80 wt%), provides a highly alkaline environment while simultaneously introducing Ca^2+^ species that promote hybrid network formation. Similarly, waste glass powders have been used as alternative soluble silicate sources, reducing reliance on high‐temperature sodium silicate production [45]. The chemical composition of aluminosilicate precursors plays a crucial role not only in determining the final geopolymer structure but also in controlling the reaction kinetics. Variations in SiO_2_ and Al_2_O_3_ content influence the dissolution rate of the precursor and the subsequent polycondensation processes. Moreover, the presence of CaO‐rich phases (e.g., slag) can significantly accelerate reaction kinetics and promote the formation of hybrid C‐(A)‐S‐H/N‐A‐S‐H gels, leading to distinct microstructural evolution compared to low‐calcium systems.

In this regard, a summary of the raw material composition of geopolymers has been reported in literature [42, 46]. Importantly, industrial by‐products such as fly ash and slag exhibit substantial compositional variability depending on their origin and processing conditions, which directly affects parameters such as Si/Al ratio, reactivity, and gel structure. As a result, geopolymerization kinetics cannot be generalized and must be considered in relation to precursor‐specific chemistry and phase composition.

When industrial alkalis are replaced by intrinsically alkaline wastes or solid‐state activation routes, the emission contribution of activators can decrease dramatically, shifting the life‐cycle balance in favor of geopolymer systems. Conversely, when highly purified NaOH and sodium silicate are used at large dosages and combined with thermal curing, the environmental advantage over OPC narrows substantially.

The sustainability aspects discussed above highlight how geopolymer chemistry and processing influence both environmental impact and material formation. These same features support the transition from geopolymer binders as low‐carbon construction materials to their use in high‐performance and functional systems. Accordingly, the following section examines their potential and evolution as advanced functional systems.

## 4 From Performing Construction Material to Functional Systems

Geopolymers have demonstrated mechanical and durability performances comparable to, and in some cases exceeding, those of OPC systems. Compressive strengths above 60–80 MPa are routinely reported for fly ash‐ and slag‐based geopolymer concretes, with values exceeding 100 MPa in optimized systems [47]. In addition to strength, geopolymers exhibit superior resistance to acid attack due to the absence of portlandite and reduced calcium hydroxide content, which constitute the degradable constituents in OPC matrices. Long‐term immersion studies of low‐calcium geopolymer formulations in sulfuric acid environments have shown mass loss reductions up to 80% compared to OPC controls [48]. High‐temperature performance further distinguishes geopolymers from conventional cement. Structural integrity has been maintained after exposure to temperatures above 1000°C, with significantly lower microcracking compared to OPC concretes. This intrinsic thermal stability has motivated applications in refractory applications, fire‐resistant panels, and infrastructure exposed to extreme thermal gradients, including oil well cementing and aerospace facilities [49, 50].

Chloride diffusion coefficients in alkali‐activated slag systems are frequently reported to be one order of magnitude lower than in OPC analogs, reflecting denser pore structures and refined gel networks [32]. In addition, long‐term durability remains a critical factor for the practical implementation of geopolymer materials. Several degradation mechanisms have been identified, including carbonation, sulfate attack, alkali exposure, and freeze–thaw cycling. These processes may affect both the chemical stability of the binding phases and the microstructural integrity over extended service times. In particular, carbonation can modify pore solution chemistry and induce structural changes, while sulfate attack may lead to expansion and microcracking. Resistance to freeze–thaw cycles and aggressive chemical environments is strongly governed by pore structure, permeability, and transport properties. Therefore, understanding these degradation mechanisms is essential for assessing long‐term performance and reliability in real‐world applications.

**FIGURE 1:**
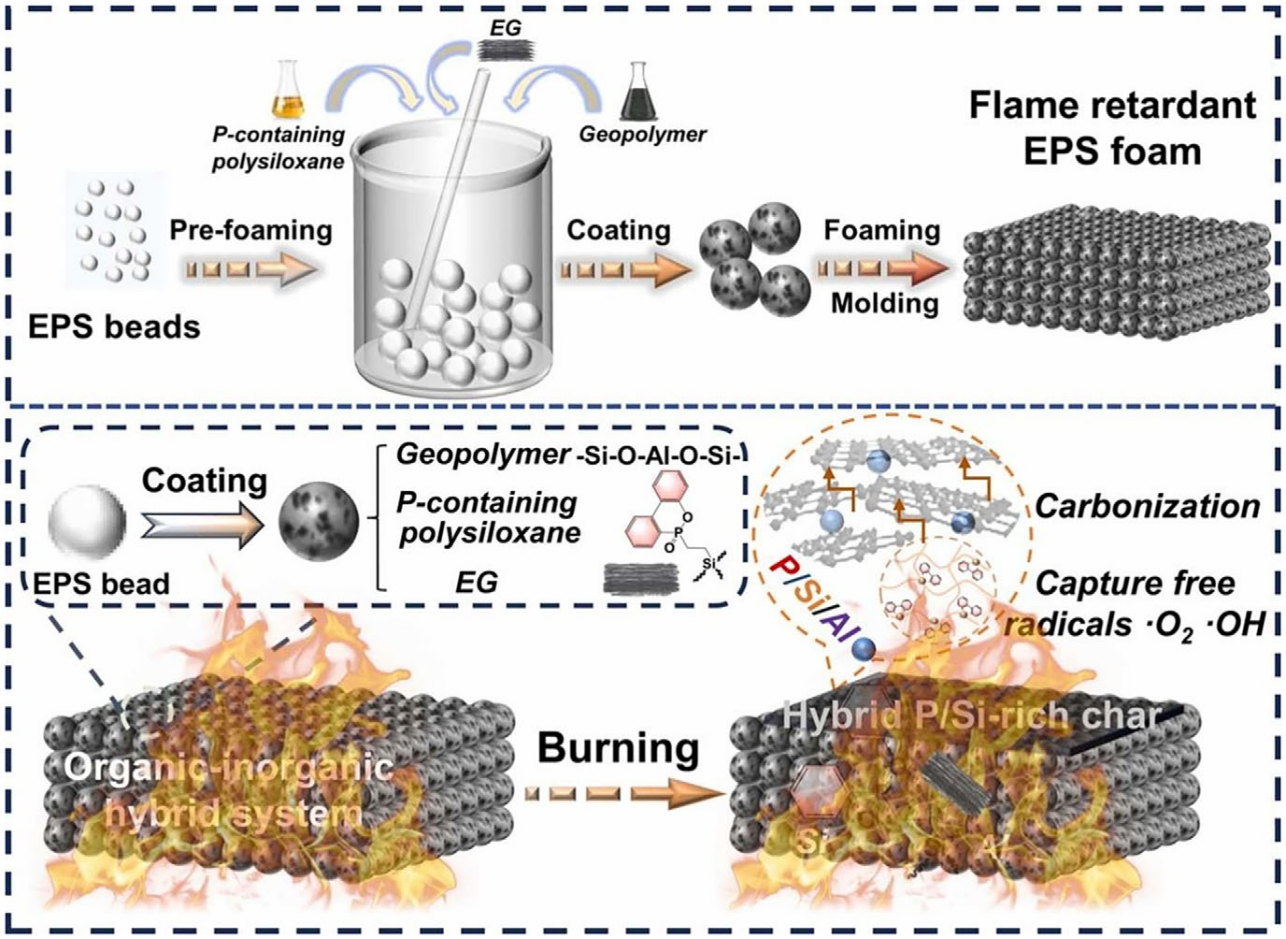
Fabrication process of EPS/PDV‐GP/EG composites and schematic diagram of flame retardancy mechanism for EPS/3.9PDV‐GP/5.0EG. Reproduced with permission [15]. Available under a CC‐BY 4.0 license. Copyright 2025, Elsevier.

By 2020, comprehensive reviews documented the technological maturity of geopolymer concrete and its increasing implementation in precast elements, pavements, and structural components [51]. Large‐scale pilot projects have demonstrated that geopolymer concrete can be manufactured, transported, and installed. However, the consolidation of geopolymer research within the construction domain has limited the perception of its broader potential. Namely, the literature has emphasized performance equivalence to OPC rather than the distinct chemical and structural tunability of alkali‐activated aluminosilicate networks. Geopolymers should not be evaluated solely as cement substitutes but as chemically programmable inorganic systems capable of supporting advanced functional applications. Their demonstrated mechanical robustness and chemical resilience provide a perspective in material multifunctionality such as adsorption, ion exchange, thermal management, and sensing that can be engineered. Recent studies are increasingly exploiting this design flexibility to develop hybrid and multifunctional systems. For example, geopolymer‐based organic–inorganic coatings have been used as flame‐retardant binders in expanded polystyrene foams, significantly improving fire resistance, thermal insulation, and mechanical performance in advanced building materials (Figure 1) [15].

## 5 Nanoengineered and Hybrid Geopolymers

### 5.1 Nanofillers and Interfacial Engineering

The incorporation of nanoscale additives into geopolymer pastes has transformed geopolymer research beyond a building material, enabling control over dissolution kinetics, nucleation density, pore refinement, and therefore multifunctionality [52]. The addition of nanosilica (typically 1–5 wt% with respect to the dry precursor) has been shown to accelerate geopolymerization by increasing the concentration of reactive silicate species and by providing heterogeneous nucleation sites. For instance, the inclusion of 2–3 wt% nano‐SiO_2_ in fly ash‐based geopolymers can increase early‐age compressive strength by 20%–40% and reduce total porosity by up to 15%. These results have been explained by considering the improvement of gel pore structure and increased polymerization degree [53]. Additionally, nanosilica modifies the effective Si/Al ratio locally, enhancing cross‐link density [25].

**FIGURE 2:**
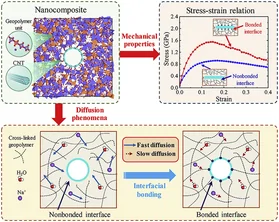
Schematic representation of the mechanism working in CNT‐geopolymer nanocomposites with nonbonded and bonded interfaces and stress–strain curves. Reproduced with permission [58]. Available under a CC‐BY 4.0 license. Copyright 2020, Elsevier.

Carbon‐based nanostructures (CBNs) introduce a distinct class of functional modifications [54]. When incorporated into the geopolymer matrix, CBNs promote microstructural refinement, resulting in lower porosity and higher yield strength. By facilitating stress transfer and limiting crack propagation, these nanostructures contribute to improved rheological response, mechanical performance, and durability in 3D‐printed geopolymer composites [55]. As a matter of fact, the presence of carbon nanotubes (CNTs) at low loadings (0.05–0.5 wt%) has been reported to improve flexural strength by 15%–30% and fracture toughness by up to 40% due to crack‐bridging mechanisms [56]. Similarly, graphene oxide additions (≤0.1 wt%) have been shown to reduce water absorption by 10%–25% while improving tensile strength [57]. These modifications demonstrate that geopolymer matrices can be engineered at the nanoscale to tailor mechanical, transport, and functional properties simultaneously. Rather than inert fillers, nanomaterials should be considered as active ingredients as they can influence the fundamental kinetics of gel formation. In fact, the rate of geopolymer gel growth increases with nucleation site density. Recent molecular dynamics simulations have demonstrated that interfacial bonding in CNT–geopolymer systems plays a dominant role in controlling structure and dynamics at the nanoscale (Figure 2). Such interactions reduce the thickness of the interphase region, limit mass transport within the matrix, and markedly increase interfacial shear strength, ultimately improving tensile behavior and delaying crack formation [58].

Controlled nanoscale heterogeneity can enhance adsorption capacity and interfacial reactivity by increasing active surface sites and microporosity. For example, nanomodified alkali‐activated systems have demonstrated 20%–50% increases in CO_2_ adsorption relative to unmodified matrices due to higher surface area and refined pore distribution [59]. Similarly, hierarchical pore structures induced by nanoscale templating improve ion transport selectivity and reduce chloride diffusion coefficients by up to one order of magnitude compared to unmodified systems [60].

However, nanomodified geopolymer systems consistently reveal the existence of an optimal additive concentration [61]. Below the percolation or nucleation threshold, improvements are modest; above critical loadings, agglomeration, viscosity increase, and incomplete dispersion lead to performance loss. For nanosilica, concentrations above ~5 wt% often result in workability reduction and strength stagnation due to particle clustering. Similarly, CNT loadings beyond the electrical percolation threshold can cause dispersion instability and reduced mechanical gain. This nonlinear behavior clearly indicates the essential chemical nanostructural design, guided by interfacial chemistry, dispersion thermodynamics, and kinetic modeling, rather than empirical optimization.

Beyond nanosilica and CNTs, several other nanostructured additives have recently been explored to tailor geopolymer properties. Nano‐Al_2_O_3_ and graphite have been shown to enhance mechanical performance through improved particle packing and network densification [62]. Nano‐ZnO, often combined with polymeric fibers such as poly(vinyl alcohol), can increase the degree of geopolymerization and promote the immobilization of hazardous species such as Cr(VI). In parallel, nano‐CuO supported on porous geopolymer foams has been used as an active catalytic phase for advanced oxidation processes, enabling efficient degradation of organic pollutants [63]. These examples illustrate the growing diversity of nanofillers employed to modulate microstructure, reactivity, and functionality in geopolymer matrices.

## 6 Halloysite‐Directed Geopolymerization: Tuning Structure and Function Across Length Scales

Halloysite (Al_2_Si_2_O_5_(OH)_4_·nH_2_O) is a naturally occurring aluminosilicate that commonly forms tubular structures (halloysite nanotubes [HNTs]) with inner diameters of 10–20 nm and lengths to micrometers [64, 65]. It shares chemical similarity with kaolinite but differs structurally due to its hollow tubular morphology and interlayer water [66, 67]. The intrinsic difference between inner (alumina‐rich) and outer (silica‐rich) surfaces, combined with their hollow tubular morphology, makes them uniquely suited as both nanoadditives and nanocarrier for controlled loading/release of active molecules [68, 69, 70, 71]. Consequently, HNTs have been explored in a wide range of fields, including biotechnology, environmental science, catalysis, and industrial applications [72, 73, 74, 75, 76, 77, 78].

HNTs introduce an interesting degree of multiscale control in geopolymer systems. Unlike conventional aluminosilicate precursors that act primarily as bulk reactive sources, halloysite combines (i) nanotubular morphology, (ii) distinct inner/outer surface chemistry, (iii) tunable reactivity upon calcination, and (iv) interfacial activity in multiphase systems. As a result, halloysite‐directed geopolymerization can result in a structural programmability that extends the applications and performances of geopolymers. This section reports HNTs function as reactive aluminosilicate precursors, rheological structuration agents, and interfacial stabilizers for phase‐change microdomains that open to the development of thermal energy storage materials.

### 6.1 Halloysite as Reactive Aluminosilicate Precursor and Rheology Control Agent

Halloysite transforms into meta‐halloysite (M‐HNT) upon calcination (typically 650°C–800°C), an amorphous aluminosilicate phase with enhanced pozzolanic reactivity. As a result, the microstructure and performance of halloysite‐based geopolymers are strongly influenced by this precalcination step, with about 750°C considered the optimal temperature to achieve a high degree of dehydroxylation and maximum reactivity [79].

Comparative studies between calcinated platy kaolin (metakaolin, M–K) and tubular meta‐halloysite demonstrate that M‐HNT often exhibits higher effective reactivity, particularly when particle size and amorphous fraction are considered [80, 81]. The amorphous content of M‐HNT reached ca. 72%, compared to ca. 64% for M–K, while the particle size of M‐HNT (ca. 6 µm) was nearly an order of magnitude lower than that of M‐K (ca. 50 µm). Isothermal calorimetry revealed higher cumulative heat release for M‐HNT‐based systems, indicating accelerated dissolution and polycondensation kinetics [80].

From a structural point of view, calcination induces coordination changes in aluminum species within HNT. Solid‐state ^27^Al MAS nuclear magnetic resonance studies on halloysite‐derived materials show transformation from six‐coordinated Al in pristine HNT to a distribution of four‐, five‐, and six‐coordinated species in M‐HNT [82]. This coordinative disorder increases the availability of reactive Al(IV) s during alkaline activation, facilitating the formation of Si–O–Al bonds and enhancing cross‐link density in the resulting geopolymeric gel.

**FIGURE 3:**
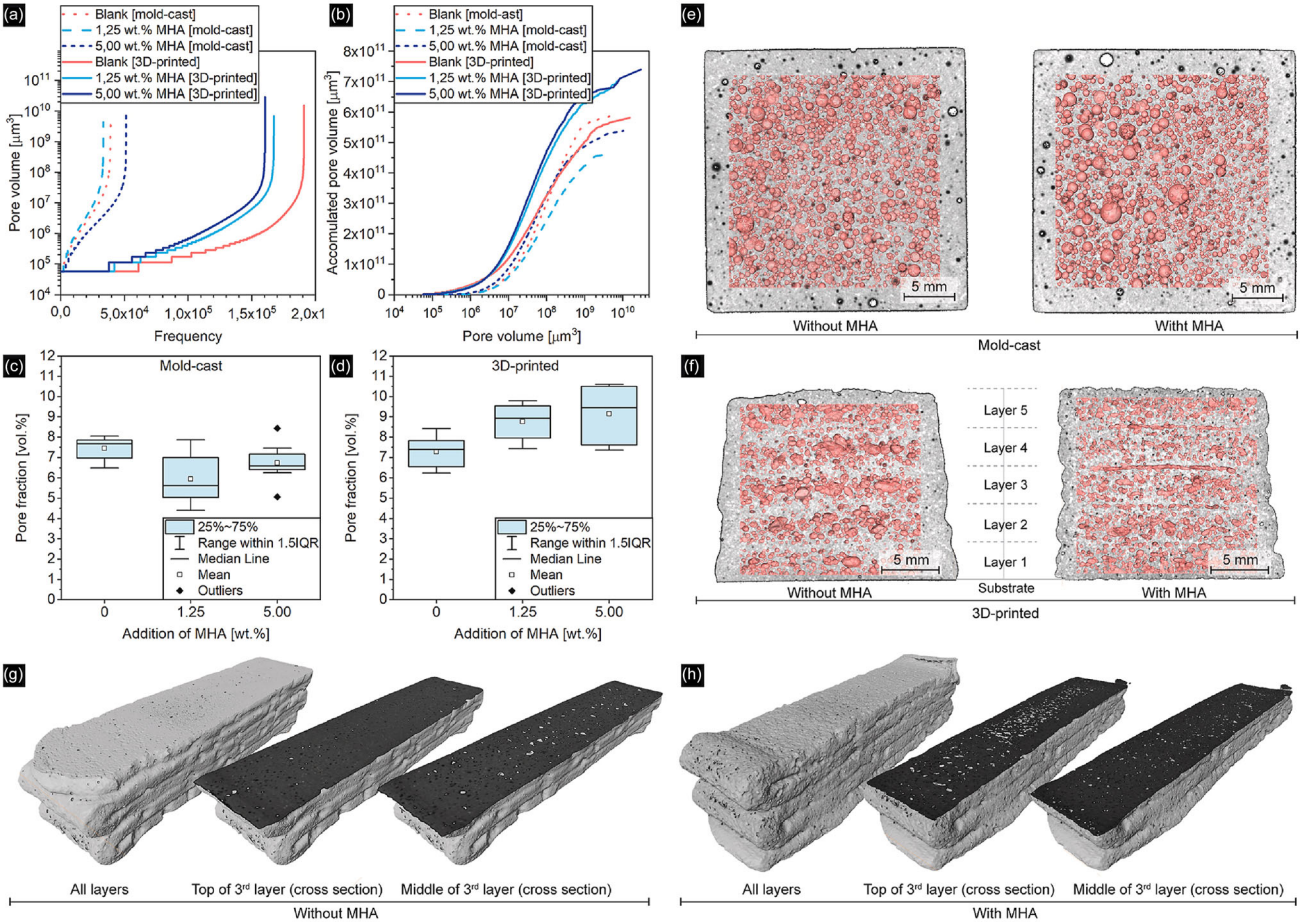
X‐ray μ‐CT results for geopolymer composites containing a different mass of M‐HNT; (a) pore volume as a function of frequency; (b) pore volume distribution; (c,d) total porosity in mold‐cast and 3D‐printed specimens; (e,f) visualization of pore distributions across the depth of mold‐cast and 3D‐printed specimens; and (g,h) interlayer defects. Reproduced with permission [83]. Copyright 2023, Elsevier.

Consequently, halloysite should not be regarded as a conventional clay filler as it behaves as a precursor whose dissolution kinetics and structural rearrangements substantially differ from those of platy kaolin. M‐HNT acts as a nucleation seed for geopolymer gel formation. Its high amorphous content (≈ 92% in some systems) accelerates early‐stage gelation, increasing buildability while shortening setting time [83]. Beyond its precursor role, HNTs influence the rheology of geopolymer pastes in the precuring time. New advances in digital manufacturing, including extrusion‐based 3D printing, geopolymer foams, and composite formulations, further illustrate how rheology control and multiscale design enable new processing routes. Recent work on 3D‐printable geopolymers has shown that small additions (1–2 wt%) of halloysite significantly increase storage modulus (G′), yield stress, and thixotropic recovery [83]. However, although low filler contents can reduce pore size and overall porosity, the associated increase in rheological parameters may hinder castability and lead to additional process‐induced porosity during molding or compaction, which can partially offset the mechanical benefits, especially at higher reinforcement levels (Figure 3). These findings have been explained by considering the peculiar morphology of halloysite, owing to its high aspect ratio and surface hydroxyl groups, that increases interparticle friction and water adsorption, enhancing strength and shape retention.

Similarly, it was reported that HNT aqueous dispersions may undergo gel formation if high aspect ratio, namely long nanotubes, are considered from a proper source [84].

Another demonstration of halloysite‐enabled nanoarchitectural control in geopolymer systems was reported by Lvov and co‐workers, who developed an electrostatically driven layer‐by‐layer (LbL) nanoencapsulation strategy for fly ash microparticles [19]. In this approach, HNTs were sequentially assembled with polyelectrolytes of opposite charge onto fly ash microcores, forming composite shells of controlled thickness (from 40 to 50 nm). This nanoscale coating architecture significantly altered geopolymer rheology and setting kinetics. Notably, LbL halloysite‐coated fly ash extended mortar setting time by approximately from 15–20 min to 70–90 min without strength loss, addressing one of the main practical limitations of geopolymer binders in high‐temperature or oil‐well environments. The claimed mechanism is interfacial: the halloysite shell retards alkaline attack, modulating the gelation step that controls geopolymer hardening [19].

This duality is critical: halloysite enables independent tuning of rheological control and chemical reactivity. In extrusion‐based additive manufacturing, such control expands the printable possibilities, improving structural stability without excessive activator adjustment.

### 6.2 Microencapsulation in Geopolymer Networks and Thermal Scaffolds

An additional halloysite role in geopolymeric composites is about its interfacial functionality. It is well established that HNTs are Pickering stabilizers to generate wax‐in‐water emulsions, which upon drying yielded spherical wax microparticles with diameters in the 3–5 µm range coated by HNTs [85, 86].

**FIGURE 4:**
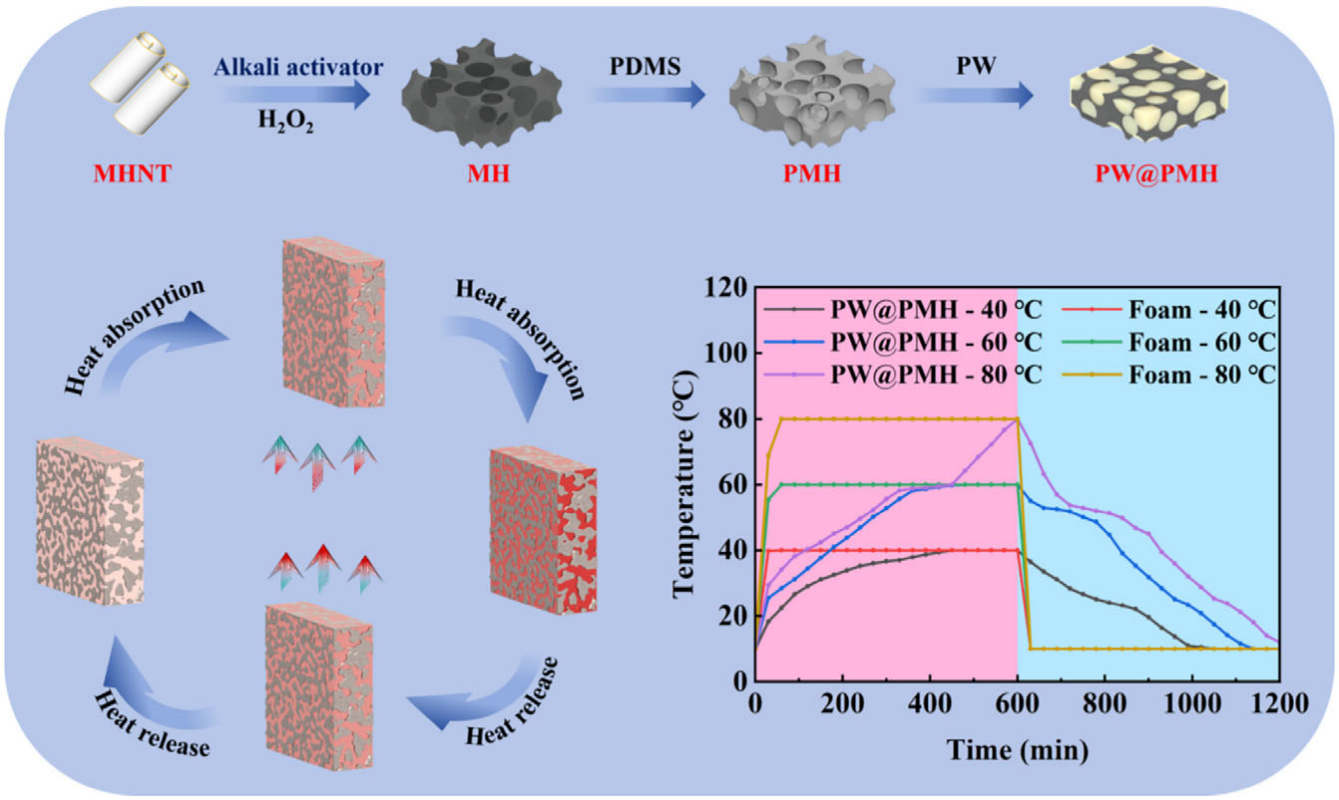
Preparation process of PMH foamed geopolymer and PW@PMH composite and schematic diagram of the thermal management of PW@PMH with the comparison of the indoor temperature under heating and cooling with and without PW@PMH. Reproduced with permission [82]. Available under a CC‐BY 4.0 license. Copyright 2026, Elsevier.

These microwax objects were embedded into an HNT‐based geopolymeric matrix, creating a composite in which wax is a uniformly dispersed microphase within the inorganic matrix. Thermogravimetric analysis indicated that the microwax‐containing geopolymer incorporated only 0.06 wt% wax as phase change material (PCM). Notwithstanding, this extremely low PCM fraction produced a step‐change in mechanical energy dissipation: the stored energy up to breaking increased by a factor of ca. 5 relative to the unfilled geopolymer, accompanied by enhanced deformability (ultimate elongation increased, while elastic modulus was reduced) [85]. It should be noted that such a low loading is far below what is normally required to measurably alter mechanical or thermal behavior in PCM–cementitious systems [87, 88, 89]. In parallel, microencapsulation of wax affected the internal architecture of the geopolymer itself: helium pycnometry showed a pronounced reduction in closed porosity from ~49.9% (neat geopolymer) to ~24.8% (microwax‐filled). The quantitative contrast with bulk‐wax loading is instructive. In the same work, comparable enhancements in flexibility and heat absorption demanded much larger wax contents, and wax filling requires contents roughly one order of magnitude larger to reach performances comparable to the microwax approach [85]. While the Pickering strategy illustrates microencapsulation as a microinterfacial dispersion issue, a complementary route employs halloysite‐derived geopolymers as hierarchical porous scaffolds for PCM encapsulation at meso/macroscales. Meta‐halloysite (M‐HNT) in the presence of hollow glass spheres (templating internal voids) and H_2_O_2_ (foaming agent), followed by poly(dimethylsiloxane) (PDMS) vapor deposition, was used to obtain a superhydrophobic foamed geopolymer (PMH) (Figure 4) [82]. The obtained geopolymer exhibits a high porosity of ca. 71% and a high specific surface area of 42 m^2^ g^−1^, with a multimodal pore hierarchy: large pores (ca. 118 µm) formed by oxygen bubbles and mid‐sized spherical voids (ca. 12 µm) generated by hollow glass sphere templating, plus nanoscale pores (in the 50–75 nm) arising from geopolymerization‐derived microstructure. When paraffin wax is vacuum‐impregnated into this halloysite‐derived geopolymer, the resulting composite reaches an encapsulation efficiency of 67%, with a melting enthalpy (ΔH_m_) of 131.1 J g^−1^ that remains essentially unaltered after 1000 heating/freezing cycles. This performance is due to superhydrophobicity related to PDMS modification and accelerates oil uptake. Halloysite is playing the key role in removing the polarity mismatch between hydrophilic aluminosilicate frameworks and hydrophobic organic PCMs that represent the main bottlenecks in PCM–geopolymer composites [82]. Moving beyond the encapsulation of organic phase‐change materials, these matrices can also serve as effective hosts for inorganic functional species. For instance, halloysite‐based geopolymers have been successfully functionalized as cost‐effective and stable phosphor hosts. By exploiting their ion‐exchange properties, trivalent rare earth ions such as Sm^3+^ and Eu^3+^ can be incorporated directly into the halloysite‐derived structure, resulting in materials that exhibit characteristic 4f–4f photoluminescence with higher intensity compared to chemosynthetic aluminosilicate hosts [90, 91].

In general, both latent heat and encapsulation efficiency are notably high for HNT‐based geopolymer as PCM carriers, when compared with microcapsules into a conventional geopolymer slurry. For instance, a PCM encapsulation efficiency of only ~32.38% and latent heat of ~66.01 J g^−1^ is reported for a graphite‐modified geopolymer [87]. More recently, latent heat around 127 J g^−1^ for shape‐stable paraffin in a porous geopolymer carrier [88], but does not show resistance and 1000‐cycle stability demonstrated in halloysite‐based systems. Even in gypsum or cementitious matrices, microencapsulated PCMs typically require significant volume fractions to deliver meaningful thermal buffering, for example, microencapsulated paraffin/gypsum building materials have been explored [92], where thermal gains must be balanced against reductions in mechanical strength and changes in workability because of higher capsule contents. Therefore, the halloysite–geopolymer systems above highlight an alternative: rather than simply increasing PCM quantity, one can increase interfacial efficiency either by (i) creating clay‐stabilized microwax particles that disperse homogeneously (Pickering route) or (ii) building a hierarchically porous mineral scaffold whose surface chemistry is tuned to selectively and stably host PCM (foamed geopolymer).

### 6.3 Halloysite‐Based Geopolymers for Advanced Functional Applications

**FIGURE 5:**
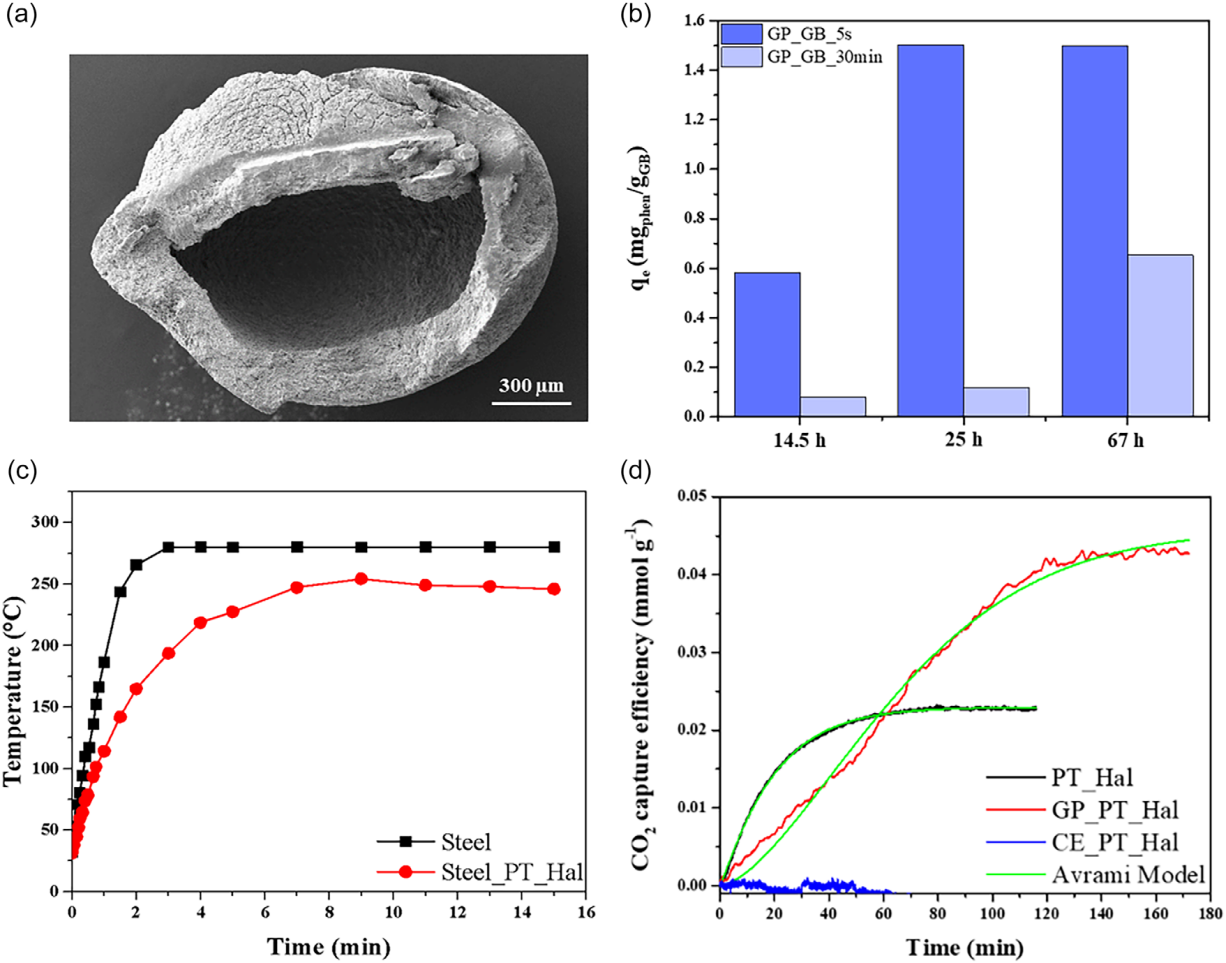
(a) SEM image of geopolymerized gel bead; (b) phenol removal efficiency of the gel beads from aqueous solution; reproduced with permission. [19] Copyright 2025, Wiley. (c) Temperature versus time profile for bare steel and the steel surface with PT_Hal to evaluate the protective effect of the film. (d) CO_2_ capture of PT_Hal‐based inorganic film and geopolymer. Reproduced with permission [20, 21]. Copyright 2024, Elsevier.

Halloysite‐based geopolymers have been extended toward environmental remediation. Starting from alginate/HNT composite beads via brief alkaline treatment (5 s), we obtained hollow spherical geopolymeric capsules [21]. Scanning electron microscope (SEM) analysis revealed that geopolymerization transformed compact gel beads into hollow capsules with internal cavities of ca. 950 μm and external porous surfaces with average pore size in the μm scale (Figure 5). This morphological transformation altered mechanical and adsorption properties. The Young's modulus decreased by 54%–76% depending on treatment time, while water uptake increased to ~26% for the short‐treated geopolymerized beads. These hollow geopolymer capsules exhibited adsorption: dodecane uptake reached ca. 38% at equilibrium and even more notably, CO_2_ capture efficiency doubled after geopolymerization, with equilibrium adsorption increasing up to 0.082 mmol g^−1^ (Figure 5b–d). These enhancements arise from the synergistic combination of internal cavity formation, increased porosity, and aluminosilicate network generation. Here, halloysite plays a dual role: (i) structural scaffold within the gel bead and (ii) reactive source for geopolymer formation, enabling a transformation from biopolymer composite to inorganic hollow adsorbent. In addition to CO_2_ and phenol, halloysite‐based geopolymers have also been explored for the removal of heavy metals highlighting the adaptability of these systems toward a broader range of environmental contaminants. Amine‐functionalized HNTs embedded in geopolymer matrices have shown efficient uptake of chromium and arsenic [93], while other studies indicate that geopolymerization can modulate the pH‐dependent leaching of heavy metals, even though complete immobilization is not always achieved [94]. A complementary functional direction was demonstrated using very long Patch HNTs (up to 30 μm in length) that were self‐assembled into fully inorganic, fibrous films without polymeric binders [20]. The entangled “bird nest” structure of these nanotubes generated self‐standing films with thicknesses ranging from tenth to hundreds of μm. The thickest films exhibited a Young's modulus of ~1710 MPa and stored mechanical energy of ~650 kJ m^−3^. Importantly, these films were intrinsically flame‐resistant due to their inorganic composition, preventing flame propagation and reducing metal substrate temperature by up to ~40°C under direct exposure. As a matter of fact, the presence of the protective film slows down the heating rate of the steel, as reflected by the reduced slope of the temperature versus time curve (Figure 5c). Subsequent alkaline activation converted the films into porous geopolymers with enhanced CO_2_ adsorption, consistent with porosity increase observed by SEM. Finally, halloysite‐based geopolymer has been successfully used in thin coatings for metal protection [95]. Wettability behavior was controlled by embedding microwax particles stabilized in HNT within the geopolymeric layer, namely the pure geopolymer coating rendered steel highly hydrophilic (contact angle 8.1°), whereas incorporation of wax/HNT microparticles increased hydrophobicity to 83.6°. This study demonstrates that halloysite‐based geopolymers can be tailored at the microinterfacial level to regulate surface wettability, opening perspectives for corrosion resistance and self‐cleaning surfaces.

## 7 Conclusions and Perspectives

Geopolymers have evolved from alternative binders into a broader class of chemically tunable inorganic polymers, whose properties arise from activation chemistry, network connectivity, and multiscale structural control. Their large‐scale application as construction materials still requires substantial research to identify truly cost‐effective and low‐carbon pathways. In particular, the environmental and economic impact of alkaline activators remains the central bottleneck. The decarbonization or replacement of sodium silicate and sodium hydroxide through waste‐derived, solid‐state, or low‐energy activation strategies is not a marginal optimization but a prerequisite for geopolymer technology to compete realistically with Portland cement in infrastructure‐scale applications. Equally important are ambient‐curing protocols, raw material standardization, and harmonized LCA methodologies that can provide transparent sustainability.

Beyond technical and environmental aspects, the large‐scale deployment of geopolymers is also limited by regulatory, market, and supply chain challenges. The lack of standardized specifications and consistent raw material supply complicates industrial implementation. Therefore, successful technology transfer will require not only advances in materials design but also the development of clear standards and reliable supply chains. At the same time, the future of geopolymers should not be restricted to bulk construction. Their intrinsic chemical tunability, thermal stability, interfacial versatility, and compatibility with nano‐ and hybrid architectures open perspectives in high‐technology domains where performance compensates material cost. Applications in adsorption and CO_2_ capture, thermal energy storage, flame‐resistant coatings, corrosion protection, and multifunctional composites illustrate how geopolymeric matrices can integrate structural robustness with advanced functionality. In these contexts, the challenge is achieving predictable control over disorder, morphology, and interfacial chemistry to design targeted properties. Thus, two parallel development pathways emerge: one focused on sustainable, economically viable activation for mass construction, and another aimed at high‐value, performance‐driven applications. Bridging these trajectories will require deeper mechanistic understanding and scalable processing strategies. If these limitations are addressed, geopolymers may consolidate their role both as next‐generation construction binders and as a versatile inorganic platform for advanced functional materials.

## Author Contributions

M.M.C. and L.L. were involved in investigation and writing – original draft preparation. G.C. was involved in reviewing, editing, and validation. S.M. was involved in conceptualization and supervision. G.L. was involved in funding acquisition and resources.

## Funding

This work was supported by Ministero dell'Università e della Ricerca, PRIN 2022 program ‐ CLIMA Project (grant B53C24006890001).

## Conflicts of Interest

The authors declare no conflicts of interest.

## Data Availability

Data sharing is not applicable to this article, as no datasets were generated or analyzed during the current study.
